# Research on nonstroke dementia screening and cognitive function prediction model for older people based on brain atrophy characteristics

**DOI:** 10.1002/brb3.2726

**Published:** 2022-10-24

**Authors:** Wei Zhang, Xiaoran Zheng, Renren Li, Meng Liu, Weixin Xiao, Lihe Huang, Feiyang Xu, Ningxin Dong, Yunxia Li

**Affiliations:** ^1^ Department of Neurology, Tongji Hospital, School of Medicine Tongji University Shanghai China; ^2^ Research Center for Ageing Language and Care at Tongji University Shanghai China; ^3^ iFlytek Research iFlytek Co. Ltd Hefei China; ^4^ Department of Radiology Tongji Hospital, School of Medicine, Tongji University Shanghai China

**Keywords:** brain atrophy, dementia prediction, eXtreme Gradient Boosting, machine learning

## Abstract

**Background:**

Brain atrophy is an important feature in dementia and is meaningful to explore a brain atrophy model to predict dementia. Using machine learning algorithm to establish a dementia model and cognitive function model based on brain atrophy characteristics is unstoppable.

**Method:**

We acquired 157 dementia and 156 normal old people.s clinical information and MRI data, which contains 44 brain atrophy features, including visual scale assessment of brain atrophy and multiple linear measurement indexes and brain atrophy index. Five machine learning models were used to establish prediction models for dementia, general cognition, and subcognitive domains.

**Results:**

The extreme Gradient Boosting (XGBoost) model had the best effect in predicting dementia, with a sensitivity of 0.645, a specificity of 0.839, and the area under curve (AUC) of 0.784. In this model, the important brain atrophy features for predicting dementia were temporal horn ratio, cella media index, suprasellar cistern ratio, and the thickness of the corpus callosum genu.

**Conclusion:**

For nonstroke elderly people, the machine learning model based on clinical head MRI brain atrophy features had good predictive value for dementia, general cognitive impairment, immediate memory impairment, word fluency disorder, executive dysfunction, and visualspatial disorder.

## 1 PREFACE

Dementia has been a significant cause of disability globally over the age of 65, as the world's aging population issue becomes increasingly severe ( Alzheimer's Association Report, 2021). It is of great meaning to find effective methods for screening, diagnosis, and monitoring dementia. Cognitive assessment is the most important method. However, the examination is time consuming and laborious, and it is easily affected by the evaluators and the cooperation of the patients, which will result in differences between the test results and the actual clinical situation. So, clinicians need to combine the patient's head structure imaging and other more objective examinations for comprehensive evaluation and judgment. Current studies have found that structural and morphological changes in the brain are closely related to cognitive decline (McInnes et al., 2017). An increasing number of studies have found that brain atrophy is closely related to cognition, and many characteristics become predictors of cognitive states.

Brain atrophy refers to reduced brain capacity due to the loss of nerve cells. It has some signs from thinner cerebral gyrus, wider, and deeper cerebral sulcus in neuroimaging. At present, it is generally believed that brain atrophy can lead to cognitive impairment such as aphasia, memory, and executive dysfunction. It occurs 10 years before cognitive impairment is detectable (Tondelli et al., 2012), and it is accompanied by the whole process of human brain aging and in different diseases. There are various patterns of brain atrophy, the whole‐brain atrophy pattern, the localized pattern atrophy, or the cortical and subcortical atrophy pattern. The correlation between atrophy pattern and dementia type also has great heterogeneity. Brain atrophy patterns do not follow the same trajectory in each type of dementia, such as Alzheimer's disease (AD), dementia with Lewy bodies, frontotemporal dementia, and vascular dementia (C. H. Li et al., 2020; B. Zhang et al., 2021). At different stages of the diseases, the pattern and progress of atrophy are also controversial. Even in the same type of dementia, the location and degree of brain atrophy have great heterogeneity in different patients (Poulakis et al., 2018). At present, it is still necessary to explore the correlation between the features of brain atrophy and cognitive impairment and discover the essential features to establish the prediction model of cognitive impairment.

There are many ways to obtain the features of brain atrophy. Most studies collected brain atrophy feature information from high‐resolution 3D sequences. However, they always base on scientific research needs and in small‐scale populations. The technical bottleneck of analysis is high and not universal. Clinical brain imaging examination is convenient and efficient, which is one of the necessary examinations for the etiological diagnosis of patients with cognitive impairment. Based on years of clinical exploration, clinicians have formed several simple, feasible, and relatively objective visual rating scales for brain atrophy assessment (Cho et al., 2009; Del Brutto et al., 2015; Koedam et al., 2011) and multiple linear measurement ratios (Uribe‐San‐Martín et al., 2018; Y. Zhang et al., 2008). However, these assessment ways are based on the specific type of dementia and a single specific assessment scale which was not comprehensive enough. Therefore, it is necessary to integrate the brain atrophy features of various assessment programs to explore the most closed brain atrophy indicators to establish dementia or cognitive domain models.

Previous studies focused on the relationship between brain atrophy and the severity of dementia, but few studies explored the relationship between brain atrophy and overall cognitive function, and the relationship between brain atrophy and the cognitive domains of word‐fluency, execution, language, and memory, and there are great controversy among these results. Oosterman et al. (2012) found that patients with severe medial temporal lobe atrophy had worse executive function. Other studies found that corpus callosum atrophy rather than medial temporal lobe atrophy in AD patients was significantly associated with executive dysfunction (Meguro et al., 2003). Some studies found that medial temporal lobe atrophy was more associated with situational memory or language dysfunction than executive dysfunction (Pleizier et al., 2012; L. Zhang et al., 2019). Therefore, the correlation between brain atrophy ratio and various cognitive domains still needs further exploration. In this study, we plan to integrate multiple high‐dimensional brain atrophy features of visual assessment and linear measurement, but routine statistical analysis has limitations in doing so. With the development of artificial intelligence and the great advantage of machine learning for multidimensional data processing, feature modeling based on multidimensional clinical data can potentially reveal the causal relationship between clinical features and diseases, which has been widely used to establish disease diagnosis and prediction models (Deo, 2015). Therefore, it is very meaningful to extract more comprehensive features of brain atrophy and to explore the predictive efficacy of dementia, cognitive function and memory, word‐fluency, execution, and visualspatial ability disorders to guide clinical diagnosis and treatment.

In this study, in order to establish a screening model that can be convenient and feasible for clinician, we extracted multidimensional brain atrophy characteristics based on the clinical MR data of nonstroke elderly patients and used machine learning method (Luo et al., 2016) to comprehensively explore the correlation between brain atrophy ratio and dementia, subcognitive domain, and general cognitive function.

## 2 MATERIALS AND METHODS

### 2.1 Inclusion and exclusion criteria

Elderly (60–85 years) who complained about memory impairment were included from January 2018 to September 2019 in the outpatient Department of Neurology and Special Clinic for Memory Impairment in Shanghai Tongji Hospital. A total 157 patients with dementia were included, and 156 normal elderly people were assessed by matching cognition of age, gender, and educational level. This study was approved by the Ethics Committee of Shanghai Tongji Hospital. All subjects were informed with a signed consent.

Diagnostic criteria for dementia: Diagnostic and Statistical Manual of Mental Disorders (4th edition, revised, DSM‐IV), 4th edition of the American Society of Psychiatry Diagnostic, and Statistical Manual of Mental Disorders. The causes of dementia included AD (*n* = 80), cerebral small vessel disease (*n* = 46), frontotemporal dementia (*n* = 2), Parkinson's disease with dementia (*n* = 2), and mixed dementia (*n* = 27). Various types of dementia use the following diagnostic criteria: (1) Cerebral small vessel disease‐related reference VASCOG (2014) (Sachdev et al., 2014) diagnostic criteria. (2) Frontotemporal dementia was diagnosed according to Gorno‐Tempini et al. (2011) diagnostic criteria. (3) Parkinson's disease with dementia: reference to the diagnostic criteria of the Movement Disorders Association (MDS) (2007) (Emre et al., 2007). (4) Alzheimer's disease reference NIA‐AA (2011) (McKhann et al., 2011) diagnostic criteria. (5) Mixed dementia that meets two or more diagnostic criteria (Skrobot et al., 2017).

Diagnostic criteria for general cognitive dysfunction: Mini‐Mental State Examination (MMSE) was used to determine the overall cognitive function. MMSE: illiterate (0) ≤ 17 points, primary school (6) ≤ 20 points, junior high school and above (9) ≤ 24 points, reference to criterion, 1990 (H. Li et al., 2016).

Diagnostic criteria for cognitive domain disorders in each subitem: Hopkins Verbal Learning Test (HVLT) was used to represent the memory function in the cognitive domain, and the immediate memory (the total score of three times) was considered to be impaired in the single cognitive domain (> 1SD). Verbal fluency test (VFT) is used to represent language function, and VFT single cognitive domain is impaired (> 1SD). Using Shape Trail Test A (STT A) to represent executive function, STT, a single cognitive domain, was impaired (> 1SD). Rey‐Osterrieth complex graphics test was impaired (> 1SD).

Exclusion criteria: (1) with a history of symptomatic stroke or head MR found cerebral infarction lesions, diameter greater than 20 mm; (2) have other central nervous system diseases such as infection, epilepsy, and demyelination history; (3) psychiatric history of schizophrenia and severe depression; (4) severe somatic diseases (such as tumor, severe kidney disease, etc.); (5) alcoholic or drug addiction; (6) obvious abnormal folic acid, vitamin B_12_, thyroid function, or syphilis antibody positive, and HIV antibody positive; (7) cannot cooperate with neuropsychological tests.

### 2.2 Clinical data collection

The general information of all subjects, including gender, age, and education level were collected. Assessment of cognitive function and daily living ability: all patients completed a set of neuropsychological tests, including the overall cognitive function assessment: MMSE. The subcognitive domain assessment included: Hopkins Verbal Learning Test (HVLT) immediate memory, 5‐min delay in memory (DeM 5 min), 20‐min delay in memory (DeM 20 min), Logical Memory‐W Story (LM), VFT, Boston Naming (BNA), STT A, and Shape Trail Test B (STT B), and Rey‐Osterrich complex graphics test copy and memory.

#### 2.2.1 MRI data acquisition and evaluation

The Siemens Verio 3.0 T superconducting magnetic resonance and standard orthogonal head coil were used. The patient was in supine position, and the head was fixed in the head bracket as far as possible. The surrounding space was filled with sponge, and the orthogonal head coil was used. AC‐PC was used as the baseline, with a thickness of 5 mm, interval of 1 mm, and FOV of 230 mm × 230 mm for scanning. The axial, sagittal, and hippocampal oblique coronal T1 weighted sequences, axial T2 weighted sequences, axial plane fluid attenuation inversion recovery (FLAIR) sequences, and T2* sequences were obtained, respectively. The parameters were as follows: TI weighted sequence: repeat time (TR) 1530 ms, echo time (TE) 9 ms, inversion time (TI) 559 ms. T2 weighted sequence: TR 4210 ms, TE 96 ms, and TI 1800 ms. FLAIR sequence scan parameters: TI 1800 ms: TR 5000 ms, and TE 94 ms. Reverse time (TI) 1800 ms. T2* sequence scan parameters: TR 55 ms, TE 45 ms, and TI 1800 ms.

#### 2.2.2 Brain atrophy feature extraction

Global cortical atrophy (GCA) (Del Brutto et al., 2015): Assessment of frontal lobe, temporal lobe, parietal lobe, occipital lobe, hippocampus, insular lobe, central sulcus, lateral fissure based on axial T1 sequence of head MRI. The scoring rules were as follows: Grade 0: expansion of no sulcus and atrophy of cerebral lobe. Grade1: There was an open sulcus expansion in the surrounding area. Grade 2: obvious sulcus expansion with deep sulcus. Grade 3: obvious sulcus dilation with narrowing of cerebral gyrus.

Posterior atrophy (PA) (Koedam et al., 2011): Assessment of parietal lobe, posterior cingulate sulcus, precuneus, and parietal occipital sulcus based on axial T1 sequence of head MRI. Grade 0: Closed posterior cingulate sulcus, parietal occipital sulcus (no atrophy). Grade 1: posterior cingulate sulcus, parietal occipital sulcus mild expansion, parietal lobe, precuneus mild atrophy (mild sulcus expansion, no significant loss of cerebral gyrus volume). Grade 2: posterior cingulate sulcus, a large expansion of the parietal occipital sulcus, a large atrophy of the parietal lobe and precuneus (severe sulcus expansion, loss of cerebral gyrus volume). Grade 3: Conspicuous bladed apical and precuneus atrophy (severe end stage atrophy).

Medial temporal lobe atrophy (MTA) (Cho et al., 2009): Evaluation based on coronal T1 sequence. The scoring rules are as follows: Grade 0: no atrophy. Grade 1: only choroidal fissure widened. Grade 2: accompanied by lateral ventricle temporal horn enlargement. Grade 3: Moderate hippocampal volume reduction (decreased hippocampal height). Grade 4: Severe hippocampal volume reduction.

Linear measurement indexes of brain atrophy (Y. Zhang et al., 2008): the following ratio were measured on the axial T1 sequence of head MRI: maximal transversal intracranial width (MTIW), maximal longitudinal intracranial width (MLIW), maximal width of frontal skull (MWF), maximal frontal horn width (MFHW), minimal intercaudate distance (MID), distance between the choroid plexuses cm (DBCP), minimal ventricular body width (MVBD), maximal width of third ventricle (MWTV), maximal width of frontal subarachnoid space (MWFSS), precentral sulcus (PS), central sulcus (CS), the sum of the widths of the four widest sulci cm (SWFS), temporal horn diameter (THD), Suprasellar cistern width (SCW). The thickness of corpus callosum (Corpus callosum genu, Corpus callosum body, and Corpus callosum sub) was measured based on sagittal T1 sequence of head MRI (Uribe‐San‐Martín et al., 2018). The following brain atrophy ratios were calculated: Evans ratio (MFHW/MTIW), bicaudate ratio (MID/MTIW), Huckman number (MFHW+MID). Huckman ratio ((MFHW+MID)/MTIW) cella media index (MVBD/MTIW), third ventricle ratio (MWTV/MTIW), ventricle index (DBCP/MFHW), frontal horns ratio (MWFSS/MLIW), four cortical sulci ratio (SWFWS/MTIW), temporal horn ratio (THD/ MTIW), cistern ambiens ratio (WCA/MTIW), suprasellar cistern ratio (SCW/MTIW), and evaluation of imaging markers for cerebral small vessel disease (CSVD).

The scoring items include: (1) number of lacunar lesions ≥ 1; (2) the number of cerebral microbleeding (CMB) ≥ 1; (3) medium and severe degree of enlarged perivascular space (EPVS) in basal ganglia (> 10); (4) White matter hyperintensity (WMH): Periventricular Fazekas score ≥ 3 or deep score 2–3 points for 1 point. The above items scored 1 point each, and the sum of each item was the total CSVD score, 0–4 points.

#### 2.2.3 Consistency assessment

All the grade indexes and linear measurement indexes of brain atrophy were completed by two neurologists with systematic training and ensuring that intra‐class correlation coefficient (ICC) > 0.8, *p* < .01.

### 2.3 Statistical analysis

All statistical analyses were completed based on software R 3. 6. 3 version. Demographic data were calculated as means ± SD or quartile IQR. Statistical analyses were used the t‐test or Wilcoxon rank sum test. Spearman or Pearson correlation coefficient was used to analyze the correlation between brain atrophy index and cognitive function. The brain atrophy data and demographic data (age, gender, and education level) of all subjects were divided into 4/5 training set and 1/5 test set through the calculation of machine learning by R caret. The fivefold cross‐validation training models were used, and the following five machine learning algorithms were used: random forest (RF), eXtreme Gradient Boosting (XGBoost), support vector machine (SVM), gradient boosting machine (GBM) and regularized logistic regression (LR) (Lee et al., 2018). AUC, sensitivity, and specificity were used to evaluate the model. According to the importance distribution results of the features in the optimal model, the dementia screen model, the overall cognitive function, and the prediction model of each subcognitive domain are constructed. The nomogram converts each regression coefficient to 0–100 in proportion. Among them, 100 is the largest β coefficient. After adding points on each independent variable to the total score, it is converted to the probability of predicting dementia or overall cognitive impairment and subcognitive domain impairment. ROC curve is drawn to evaluate the accuracy of the model, and the calibration curve is used to verify the model.

## 3 RESULTS

### 3.1 Comparison of general information

There was no significant difference between the dementia group and the normal cognitive group in age, gender, education years, WMH, EPVS, CMB, lacunar infarction, and CSVD total score (Table 1).

**TABLE 1:** The comparison between dementia group and normal group, values are expressed as mean ± standard deviation

	Normal (*n* = 156)	Dementia (*n* = 157)	*p*
Sex (female, %)	79 (50.6)	83 (52.9)	.735
age (mean [SD])	70.32 ± 7.97	70.46 ± 8.16	.88
Education(years)	9.00 (9.00, 12.00)	9.00 (9.00, 12.00)	.485
CSVD	1.0 (1.00,2.00)	1.0 (1.00,2.00)	1.000
WMH ventricle	1.0 (1.00,2.00)	1.0 (1.00,2.00)	.144
WMH deep	1.0 (1.00,2.00)	1.0 (1.00,2.00)	.234
LI	0.00 (0.00,0.00)	0.00 (0.00,0.00)	.772
EPVS	0.00 (0.00,0.00)	0.00 (0.00,0.00)	.801
CMB	0.00 (0.00,0.00)	0.00 (0.00,0.00)	.356

Abbreviations: CMB, cerebral microbleeding; CSVD, cerebral small vessel disease; EPVS: enlarged perivascular space; LI, lacunar infarction; WMH, white matter hyperintensity.

### 3.2 Results of correlation analysis between brain atrophy and cognition

**FIGURE 1:**
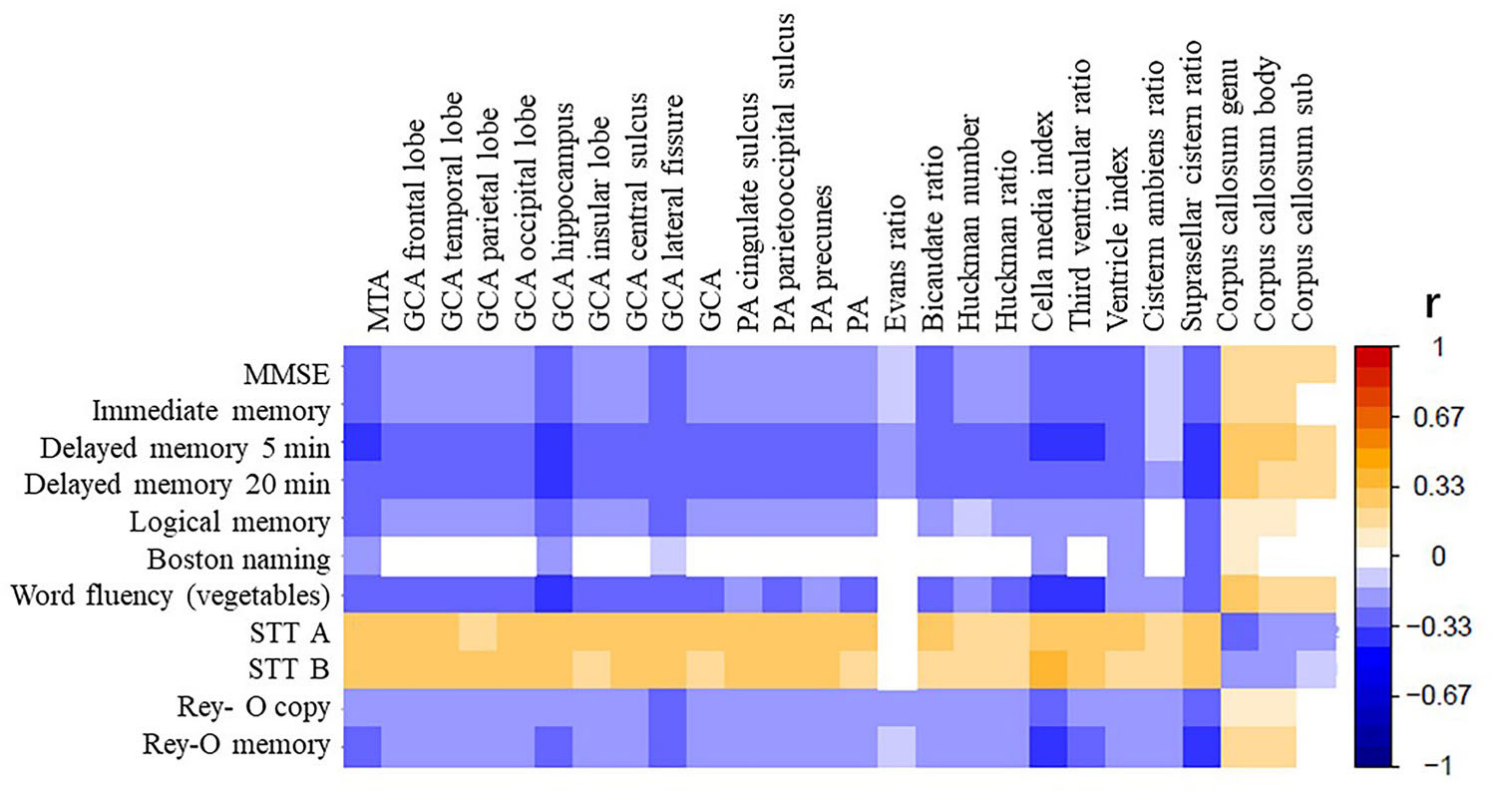
The correlation between brain atrophy and cognition Abbreviations: GCA, Global cortical atrophy; MMSE, Mini‐Mental State Examination; MTA, Medial Temporal Lobe Atrophy; PA, Posterior atrophy, STTA/B, Shape Trail Test A/B; Rey–O copy and memory, Rey‐Osterrich complex graphics test copy and memory

From the graph, the general cognitive function and subcognitive domain were correlated with multiple brain atrophy indexes. The score of MMSE, immediate memory, word fluency, and visualspatial function negatively correlated with temporal horn ratio (*p* < .01) and positively correlated with the genu of corpus callosum (*p* < .01). The positive correlation coefficient between executive function and temporal horn ratio was the largest (*r* = .351, *p* < .01), and the negative correlation coefficient between executive function and the genu of corpus callosum was the largest (*r* = −.289, *p* < .01). The detailed results are shown in Figure 1.

### 3.3 Results of dementia prediction model based on machine learning algorithm

**FIGURE 2:**
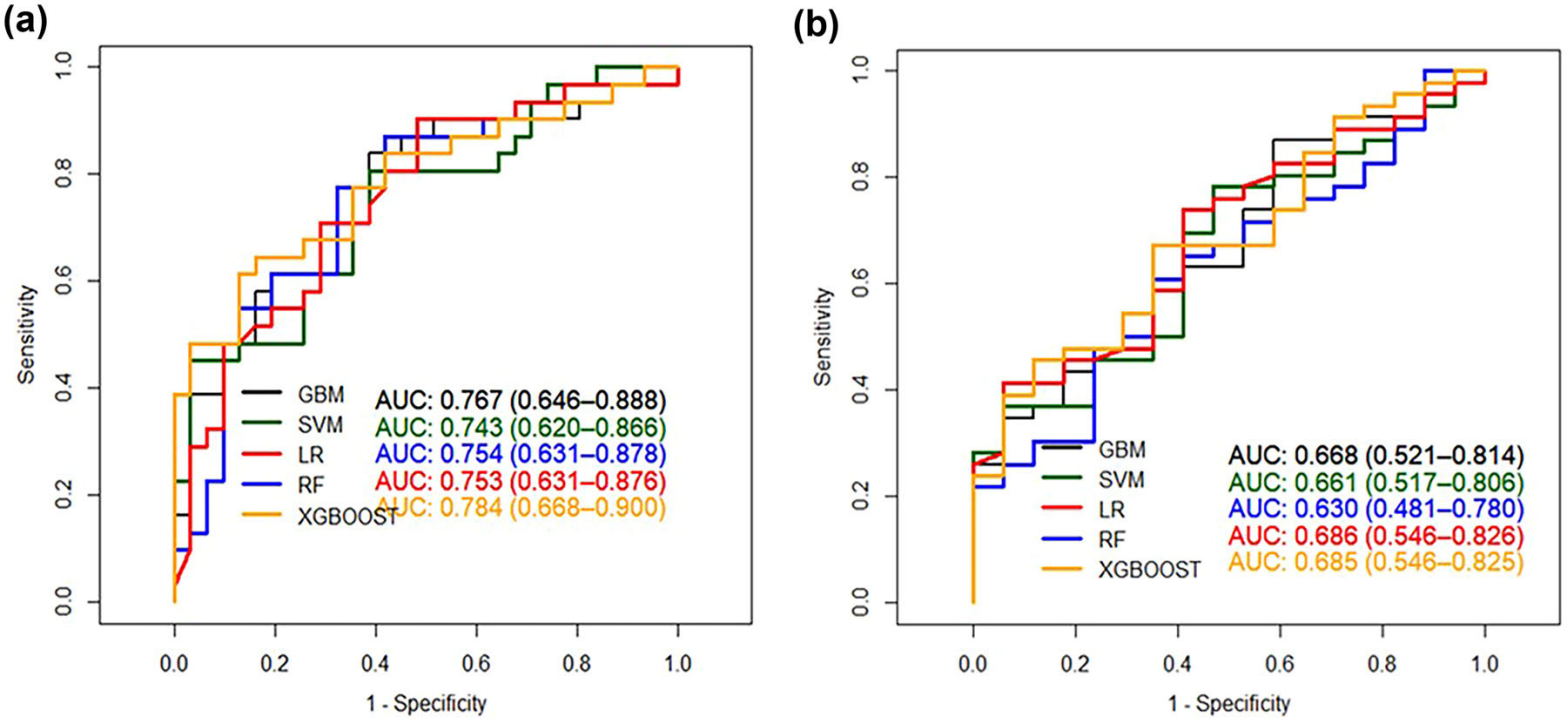
(a) The receiver operating characteristic (ROC) of five general cognition model. (b) ROC of five dementia model Abbreviations: GBM, gradient boosting machine; LR, regularized logistic regression; RF: random forest; SVM, support vector machine; XGBoost, eXtreme gradient boosting

By analyzing the characteristics of brain atrophy and clinical features of patients, XGBoost showed the best specificity (0.839) and AUC (0.784) specificity (0.839) and AUC (0.784) on the test set, and the sensitivity was 0.645. GBM showed the optimal sensitivity (0.839), specificity (0.613) and AUC (0.767) on the test set. The detailed results are shown in Table 2 (Appendix 1) and Figure 2b. The top 20 indexes of brain atrophy index predicted by GBM model were THD/MTIW, suprasellar cistern ratio, cella media index, THD, corpus callosum genu and body thickness, MTIW, DBCP, DBCP/MFH, Huckman ratio, SCW, MID/MTIW, MLIW, MWFSS / MLIW, MTA, MVBD, Evans ratio, Huckman number, MWFSS, MWTV/MTIW. The detailed results are shown in Figure 4.

### 3.4 Prediction results of general cognition and subcognitive domains based on machine learning algorithm

**FIGURE 3:**
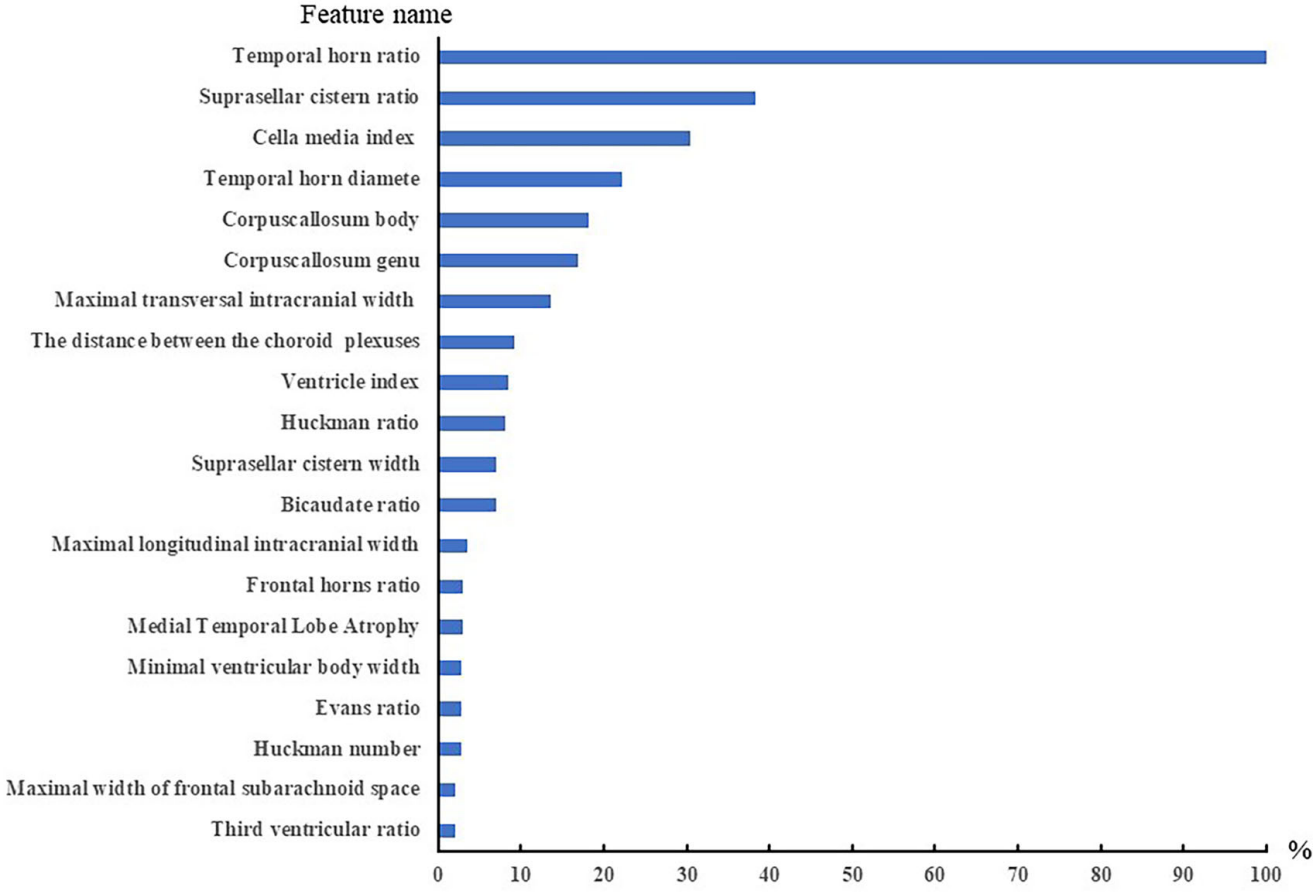
The important 20 features of dementia prediction model (Gradient Boosting Machine)

When predicting the overall cognitive impairment, the SVM model showed the optimal sensitivity (0.783), specificity (0.529) and AUC (0.661) on the test set. GBM showed the best specificity (0.941), sensitivity (0.348) and AUC (0.668). The RF model showed the best AUC 0.686, the sensitivity was 0.413, and the specificity was 0.941. The detailed results are shown in Table 3 (Appendix 1) and Figure 2a. The top 10 indexes of brain atrophy index predicted by RF model were THD/MTIW, SCW/MTIW, cella media index, THD, corpus callosum body thickness, corpus callosum genu thickness, MTIW, DBCP, DBCP/MFHW, and Huckman ratio. The detailed results are shown in Figure 3.

**FIGURE 4:**
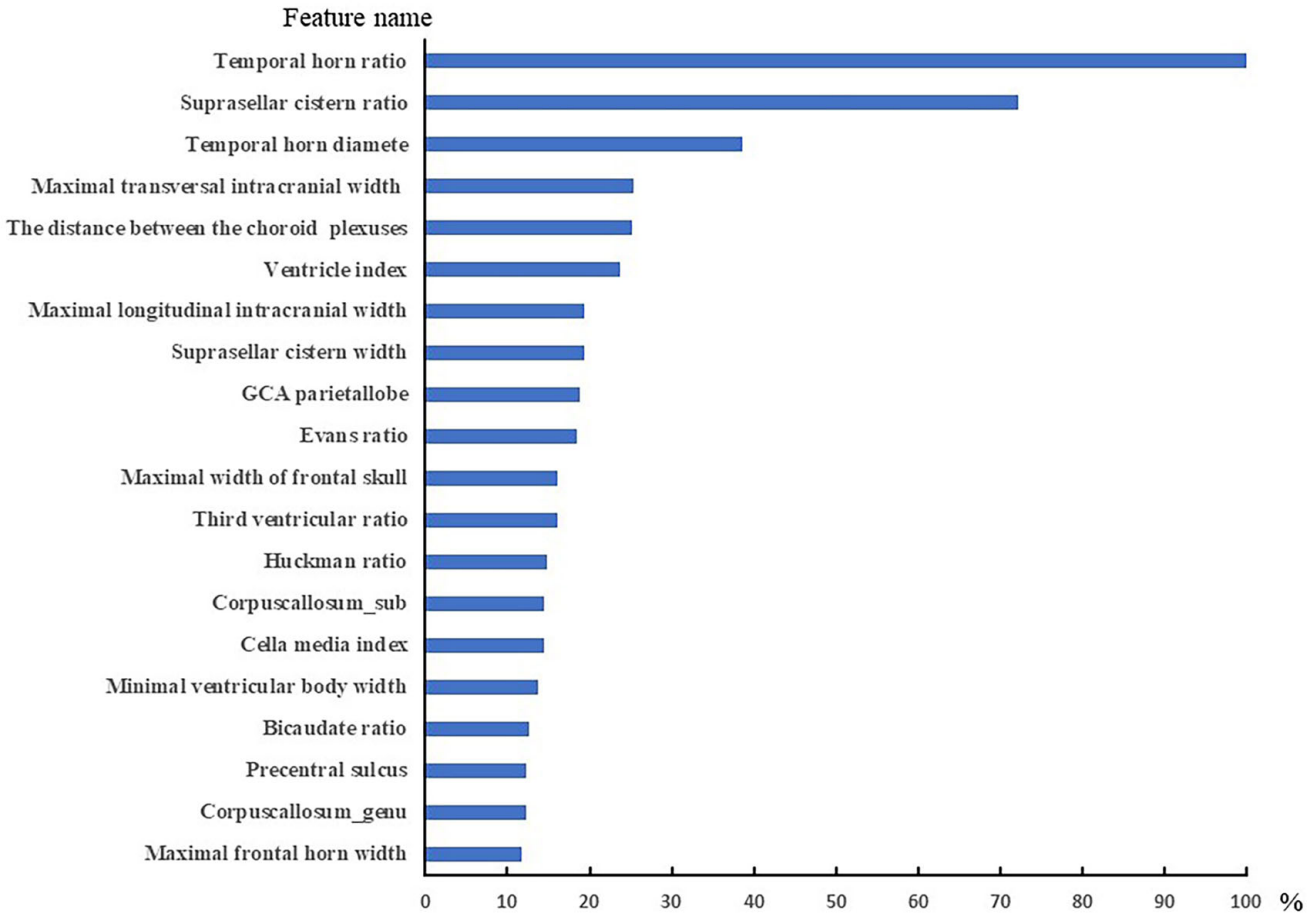
The important 20 features of general cognition prediction model (Random Forest)

**FIGURE 5:**
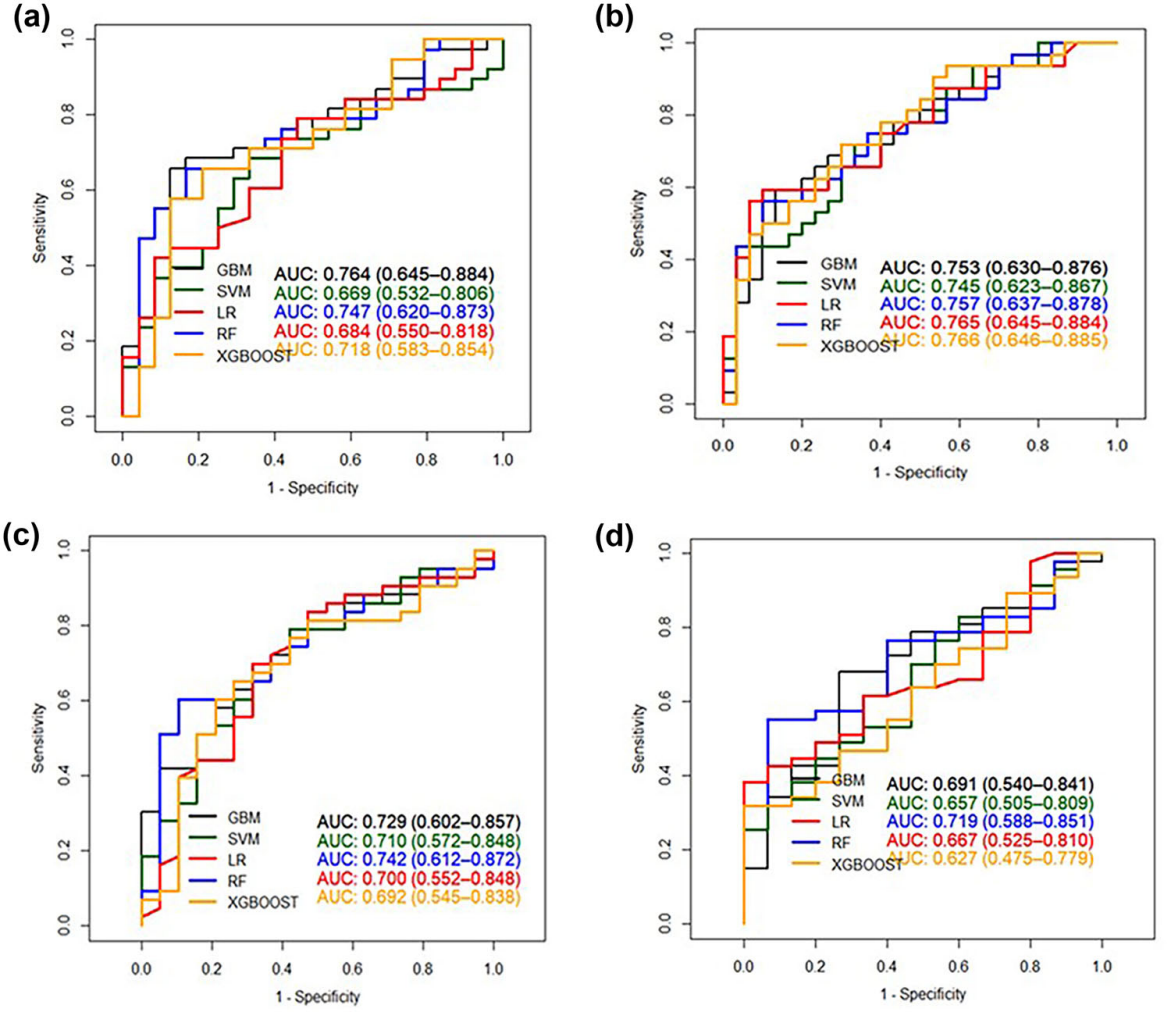
(a) The receiver operating characteristic (ROC) of five memory model. (b) The ROC of five Word‐fluency model. (c) The ROC of five execution model. (d) The ROC of five visuospatial model Abbreviations: GBM, gradient boosting machine; LR, regularized logistic regression; RF: random forest; SVM, support vector machine; XGBoost, eXtreme gradient boosting

When predicting single cognitive domain‐immediate memory impairment, SVM model has the best sensitivity (0.684), specificity (0.667), and AUC (0.669) on the test set. The test set of random forests showed the optimal specificity (0.917), sensitivity of 0.421, and AUC of 0.684. GBM showed the best AUC (0.766) on the test set, the sensitivity was 0.658, AUC was 0.875, and the details are shown in Table 4 (Appendix 1) and Figure 5a. The top 10 indicators of GBM model for predicting single immediate memory impairment were SCW/MTIW, THD/MTIW, THD, DBCP, MFHW, years of education, MTA, cella media index, corpus callosum genu thickness, and MTIW.

The XGBoost model has the best sensitivity (0.719), specificity (0.700), and AUC (0.766) when predicting single cognitive domain word fluency (vegetable). The RF model showed the best specificity (0.933), sensitivity was 0.562, AUC was 0.765, and the details are shown in Table 5 (appendix 1) and Figure 5b. The top 10 indicators of XGBoost model for predicting cognitive domain damage were SCW/MTIW, corpus callosum genu thickness, THD, cella media index, SCW, MVBD, THD/MTIW, education, age, and corpus callosum subthickness.

When predicting a single cognitive domain‐executive function, SVM model has the best sensitivity of 0.791, specificity of 0.579, and AUC of 0.710. The LR model showed the best specificity was 0.895, the sensitivity was 0.605, and the AUC was 0.742; the details are shown in Table 6 (Appendix 1) and Figure 5c. The top 10 indicators of LR model for predicting cognitive domain damage were THD/MTIW, THD, cella media index, MTA, MVBD, MID/MTIW, SCW/MTIW, GCA—hippocampal atrophy, corpus callosum genu thickness, and DBCP/MFHW.

When predicting single cognitive domain visuospatial dysfunction, GBM model showed the best sensitivity (0.681), specificity (0.733), and AUC (0.691). RF and XGBoost model showed the best specificity (1.000), RF sensitivity (0.383), and AUC (0.667). The details are shown in Table 7 (Appendix 1) and Figure 5d. LR model showed the best AUC of 0.719, the sensitivity of 0.533, and the specificity of 0.933. The top 10 indicators of LR model for predicting cognitive domain damage were SCW/MTIW, SCW, THD, THD/MTIW, GCA hippocampal atrophy, cella media index, MVBD, DBCP, MTA, and WCA.

### 3.5 Predictive efficacy of dementia, general cognition, and subcognitive domains

**FIGURE 6:**
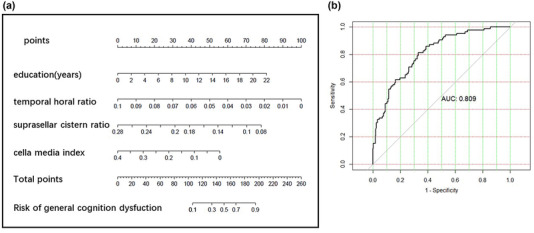
(a) The nomogram of screening general cognition. (b) The receiver operating characteristic (ROC) of the nomogram

Construction of nomogram to predict the overall cognitive impairment: Based on the important feature distribution of the random forest model, education, THD/MTIW, cella media index, and SCW/MTIW are selected to construct the screening model. The AUC of the nomogram was 0.809, the sensitivity was 0.814, and the specificity was 0.668. The details are shown in Figure 6a,b.

**FIGURE 7:**
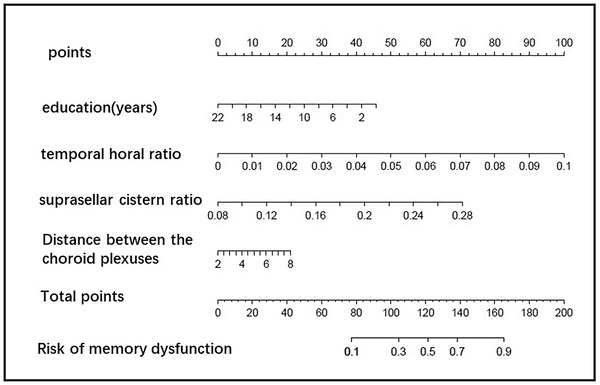
The nomogram of screening memory dysfunction

Construction of nomogram for predicting single cognitive‐immediate memory impairment: Based on the important feature distribution of GBM, education, THD/MTIW, SCW/MTIW, and the DBCP are selected to construct the model. The AUC of the nomogram was 0.780, the sensitivity was 0.726, and the specificity was 0.716. The details are shown in Figure 7.

**FIGURE 8:**
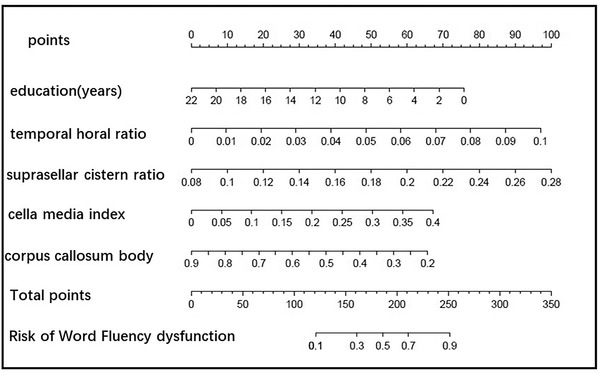
The nomogram of screening word fluency dysfunction

Construction of a nomogram for predicting single cognitive‐word fluency disorder: Based on the important feature distribution of XGBoost, education, THD/MTIW, cella media index, SCW/MTIW, and corpus callosum body thickness are selected to construct the model. The AUC of the nomogram was 0.774, the sensitivity was 0.664, and the specificity was 0.806. The details are shown in Figure 8.

**FIGURE 9:**
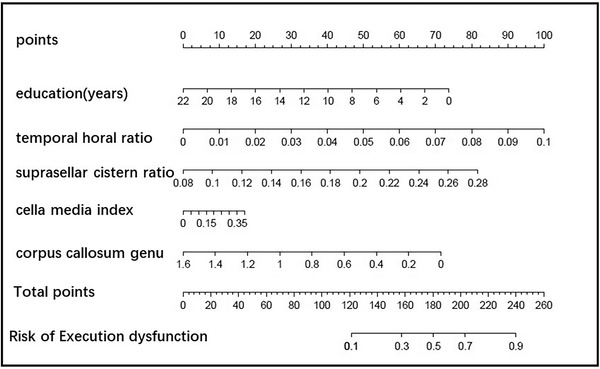
The nomogram of screening execution dysfunction

Construction of a nomogram for predicting single cognitive‐executive dysfunction. Based on the important feature distribution of LR, education, THD/MTIW, cella media index, SCW/MTIW, and the thickness of the corpus callosum genu are selected to construct the model. The AUC of the nomogram was 0.761, the sensitivity was 0.698, and the specificity was 0.726. The details are shown in Figure 9.

**FIGURE 10:**
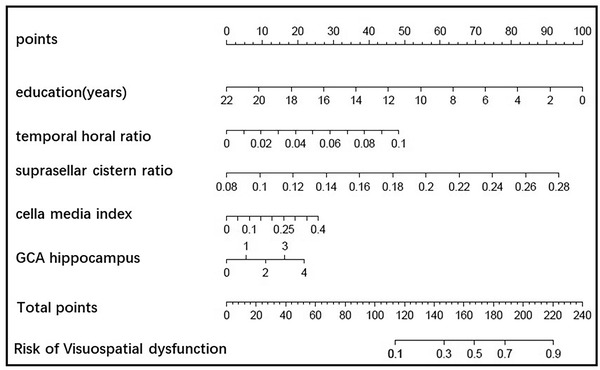
The nomogram of screening visuospatial dysfunction

Construction of the nomogram for predicting single cognitive‐visualspatial disorder: Based on the important feature distribution of LR, education, THD/MTIW cella media index, SCW/MTIW, and GCA hippocampal atrophy are selected to construct the model. The AUC of the line map was 0.770, the sensitivity was 0.844, and the specificity was 0.603, as shown in Figure 10.

**FIGURE 11:**
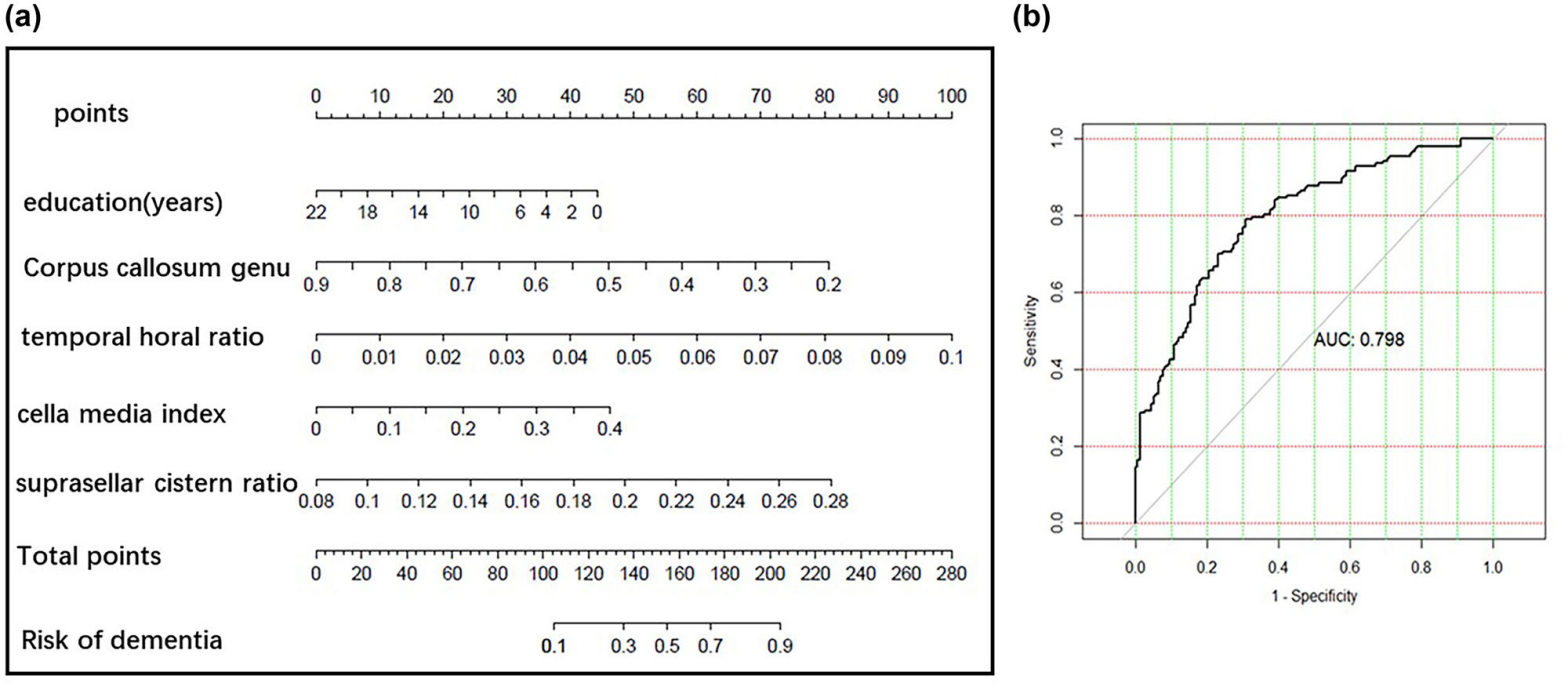
(a) The nomogram of screening dementia. (b) the receiver operating characteristic (ROC) of the nomogram

Construct a nomogram for predicting dementia: Based on the XGBoost model, the top four brain atrophy indexes are selected: THD/MTIW, cella media index, SCW/MTIW, and genu thickness of corpus callosum. Combined with education, the prediction was established. The AUC of dementia was 0.798, the sensitivity was 0.790, and the specificity was 0.692. The details are shown in Figure 11a,b.

## 4 DISCUSSION

It is urgent to establish a convenient screening tool for dementia, and brain atrophy is closely related to human brain structure and advanced cognitive function, and it is widely used as an important factor in dementia screening model. Previous studies mostly established prediction models based on high‐resolution structural images of the whole brain or magnetic resonance data of diffusion tensor imaging of 3DT1 (Lee et al., 2018; Savarraj et al., 2021; Tam et al., 2019). However, the acquisition of these features was very complicated and was not suitable for clinicians. Therefore, based on clinical routine magnetic resonance sequence, we used visual assessment and simple linear measurement features to establish the prediction model of dementia, general cognitive function, and memory, language, execution, and visuospatial domain. The model was verified by internal data and established nomogram to predict dementia, general cognitive dysfunction, and cognitive domain dysfunction in the individual level.

### 4.1 Dementia prediction model

Previous dementia prediction models are mainly based on the demographic characteristics (Hou et al., 2019; Kivipelto et al., 2006) and neuropsychological assessment scale characteristics. Due to the close correlation between dementia and brain structure and function, models established by clinical features combined with imaging features were more explanatory and high prediction efficiency. Some studies have found that brain atrophy played an important role in predicting dementia (Frenzel et al., 2019; Kivipelto et al., 2006). Grassi and Frenzel et al. established model in which sensitivity and specificity could reach more than 0.8 by incorporating cortical atrophy index into multimodal MRI feature and other features (Frenzel et al., 2019; Grassi et al., 2018). Through the Alzheimer's Disease Neuroimaging Initiative (ADNI) database, Tam et al. used machine learning method to extract characteristics of multiple subtypes of brain atrophy and neuropsychological measurement scores to establish prediction model for the conversion of MCI patients to AD within 3 years. The study found that compared with MMSE score alone, the combination of MMSE score and multimodal structural brain atrophy data could improve the prediction accuracy (Tam et al., 2019). However, it is difficult to illustrate clearly on the brain atrophy features extracted based on machine learning methods, and obtain 3D brain structure scanning data for clinicians. In some studies, models established based on clinical visual scale and linear measurement data also had excellent sensitivity and specificity. These features included temporal horn ratio, corpus callosum genu thickness, suprasellar cistern ratio, and so on. Studies varied in the extraction of brain atrophy features. Among them, temporal horn ratio, suprasellar cistern ratio, and corpus callosum genu thickness were used as predictors to establish prediction models in most researches (Frenzel et al., 2019; Grassi et al., 2018; Hou et al., 2019; Kivipelto et al., 2006; Savarraj et al., 2021). Some studies found that cella media index was significantly different between AD and normal elderly (Chrzan et al., 2019). In this study, the XGBoost machine learning model predicted dementia with AUC of 0.8, and we found that temporal horn ratio, cella media index, suprasellar cistern ratio, and the thickness of the corpus callosum genu were selected as the atrophy characteristics of dementia.

In this study, our prediction model is essentially a classifier for dementia, and four major cognitive domain disorders are based on cross‐sectional data. However, many other studies are based on the prediction model established by similar methods (Shigemizu et al., 2019; Shimoda et al., 2021), which can be used for dementia screening rather than accurate diagnosis. Considering the sample size, we did not make a more detailed classification of dementia types. The common types are AD, vascular dementia, and mixed dementia. However, according to recent studies (Reith & Mühl‐Benninghaus, 2015), the current distribution of dementia types is more coincident with that in clinical practices. We constructed a dementia screening model based on clinical practicability for the community elderly because precise classification diagnoses are difficult for community doctors.

The study found that education was closely related to cognitive impairment (Wilson et al., 2019). Stern (2006) proposed that education level was an important factor affecting cognitive reverse (CR). Some studies believed that education could improve CR, thereby enhancing individual resistance to cognitive impairment and coping with cognitive decline caused by age (Brayne et al., 2010). Through the analysis of the importance of features, this study found that education is more important in model establishment. Thus, it is included as one of the predictors. We combined the important features of brain atrophy with educational to establish dementia prediction model, AUC reaching to 0.8.

### 4.2 Prediction model of general cognitive dysfunction and subcognitive domain dysfunction

At present, few studies were involved in the models of general cognition and subcognitive domain disorders based on clinical brain atrophy features, among which most were cohort studies. A study based on 3D whole brain volume MR data found that all brain volume and local brain volume were closely related to the decline of overall cognitive function (Jokinen et al., 2020). Another study found that medial temporal lobe atrophy and subcortical atrophy could independently predict memory function and executive function decline (Jokinen et al., 2012). The atrophy of the hippocampus and olfactory cortex can predict the decline of memory function (Stoub et al., 2010). A 10‐year follow‐up study of 60 healthy elders found that hippocampal volume could predict changes in executive function (Aljondi et al., 2019). A few studies extracted the nondementia population over the age of 70 (*n* = 921) for 12 years of follow‐up by using visual scale assessment of brain atrophy (frontal lobe, parietal lobe, temporal lobe, and occipital lobe), lacunar, and WMH to establish a model. The study found that cortical atrophy was relative to cognitive decline (Sacuiu et al., 2018). Other studies found that frontal lobe atrophy could predict decreased word fluency (Söderlund et al., 2004). Our study also found a correlation between brain atrophy and cognition, which could be used to predict general cognitive function, memory, execution, language, and visualspatial subcognitive domains. However, these studies were conducted by cross‐sectional or follow‐up analysis based on partial brain atrophy data or 3DMRI data. Among them, there were few studies on the prediction of cognitive dysfunction by combining multiple brain atrophy indicators. Our study only selected multiple and relatively comprehensive measurable brain atrophy data to establish models and without any neuropsychological scale. Our models’ prediction performance in internal verification were acceptable. Moreover, we also established nomogram of general cognitive impairment and subcognitive domain impairment on the individual level, with AUC reaching to 0.8.

The machine learning methods applied in this study were all from the methods recommended in the biomedical guidelines (Luo et al., 2016). In this study, the machine learning model of XGBoost showed its best prediction effect for dementia. Since XGBoost supported parallel and distributed computing, it runs fast and can adjust multiple hyperparameters, which can effectively reduce overfitting (Shimoda et al., 2021). Moreover, the XGBoost algorithm also can standardize the regularization term to prevent the model from overfitting. Finally, the XGBoost algorithm model used can automatically screen the importance of predictive variables while ignoring the interference of irrelevant variables, which greatly improved the effectiveness of our research (Wang et al., 2022).

This algorithm also has good prediction results for single cognitive domain–word fluency. In the prediction of executive function and visual space function, the prediction results of logical regression are better. The L2 regularized logistic regression used in this study can better solve the problem of multiple collinearities. GBM has a good prediction effect in predicting single cognitive domain‐immediate memory, which may be related to the robustness of GBM for data and can effectively deal with multidimensional data.

Certainly, limitations exist. (1) This study is a single‐center study, which still needs larger multicenter data to expand the sample size, so as to train more optimized models. (2) The dementia screening model established in this study had good predictive effect in internal validation and could continue external validation in follow‐up cohort or other cohorts. (3) This study established a two‐class prediction model based on normal cognition and dementia. We need to further collect data to establish a three‐class prediction model of normal cognition, MCI, and dementia. (4) The brain atrophy evaluation index selected in this study was a linear measurement index. Although it was practical, more objective brain atrophy evaluation indexes based on clinical MRI needed to be developed.

## 5 CONCLUSIONS

For nonstroke elderly people, the machine learning model based on clinical brain MRI brain atrophy features had good predictive value for dementia, general cognitive impairment, immediate memory impairment, word fluency disorder, executive dysfunction, and visualspatial disorder. XGBoost model has the best effect in predicting dementia, temporal horn ratio, corpus callosum genu thickness, cella media index, suprasellar cistern ratio, and education are important predictors of dementia.

## FUNDING

Database Project of Tongji Hospital of Tongji University (Grant No. TJ (DB)2102); Clinical Research Project of Tongji Hospital of Tongji University (Grant No. ITJ(ZD)2002); Project of the National Social Science Foundation of China (NSSFC) (No. 19CYY018); Shanghai Hospital Development Center Foundation (Grant No. SHDC12021110)

## AUTHOR CONTRIBUTIONS

The study desgined by Wei Zhang, Xiaoran Zheng, Ningxin Dong and Yunxia Li. Material preparation, data collection and analysis performed by Wei Zhang, Xiaoran Zheng, Renren Li, Meng Liu, and Weixin Xiao. Statistical analysis was performed by Wei Zhang and Feiyang Xu. The first draft of the manuscript written by Wei Zhang and Lihe Huang and critically revised by all authors. Feiyang Xu provided technical assistance with the machine learning aspects of the project. The authors thank him for his support. All authors read and approved the final version of the manuscript.

## CONFLICT OF INTEREST

The authors declare no conflict of interest.

### PEER REVIEW

The peer review history for this article is available at https://publons.com/publon/10.1002/brb3.2726.

## Data Availability

The data that support the findings of this study is available from the corresponding author, upon reasonable request.

## APPENDIX A

### A.1

**TABLE 2:** The results of brian atrophy feature to predict general cognition of 5 machine learning algorithms

**Model**	**Set**	**Sensitivity**	**Specificity**	**AUC(95%CI)**
Gradient Boosting Machine	train	0.801	0.841	0.833(0.838‐0.927)
	test	0.348	0.941	0.668 (0.521‐0.814)
SVM	train	0.823	0.899	0.919 (0.880‐0.958)
	test	0.783	0.529	0.661(0.517‐0.806)
Regularized Logistic Regression	train	0.753	0.783	0.826 (0.768‐0.884)
	test	0.609	0.647	0.630(0.481‐0.780)
Random Forest	train	1.000	1.000	1.000
	test	0.413	0.941	0.686(0.546‐0.826)
XBGBoost	train	0.892	0.870	0.952(0.928‐0.976)
	test	0.457	0.882	0.685(0.546‐0.825)

**TABLE 3:** The results of brian atrophy feature to predict the dementia of 5 machine learning algorithms

**Model**	**Set**	**Sensitivity**	**Specificity**	**AUC(95%CI)**
Gradient Boosting Machine	train	0.808	0.778	0.880(0.839‐0.920)
	test	0.839	0.613	0.767(0.646‐0.888)
SVM	train	0.848	0.794	0.878(0.835‐0.922)
	test	0.806	0.613	0.743(0.620‐0.866)
Regularized Logistic Regression	train	0.744	0.865	0.867(0.822‐0.911)
	test	0.774	0.677	0.754(0.631‐0.878)
Random Forest	train	1.000	1.000	1.000
	test	0.839	0.516	0.724(0.596‐0.852)
XGBoost	train	0.896	0.817	0.911(0.876‐0.947)
	test	0.645	0.839	0.784(0.668‐0.900)

**TABLE 4:** The results of brian atrophy feature to predict the memory of 5 machine learning algorithms

**Model**	**Set**	**Sensitivity**	**Specificity**	**AUC(95%CI)**
Gradient Boosting Machine	train	0.770	0.730	0.824(0.772‐0.876)
	test	0.658	0.875	0.764(0.645‐0.884)
SVM	train	0.645	0.810	0.787(0.728‐0.845)
	test	0.684	0.667	0.669(0.532‐0.806)
Regularized Logistic Regression	train	0.632	0.800	0.785(0.728‐0.841)
	test	0.658	0.833	0.747(0.620‐0.873)
Random Forest	train	1.000	1.000	1.000
	test	0.421	0.917	0.684(0.550‐0.818)
XGBoost	train	0.783	0.910	0.914(0.879‐0.948)
	test	0.579	0.875	0.718(0.583‐0.854)

**TABLE 5:** The results of brian atrophy feature to predict the word‐fluency of 5 machine learning algorithms

**Model**	**Set**	**Sensitivity**	**Specificity**	**AUC(95%CI)**
Gradient Boosting Machine	train	0.758	0.836	0.861(0.816‐0.906)
	test	0.594	0.867	0.753(0.630‐0.876)
SVM	train	0.705	0.820	0.839(0.790‐0.887)
	test	0.719	0.667	0.745(0.623‐0.867)
Regularized Logistic Regression	train	0.826	0.672	0.783(0.725‐0.840)
	test	0.562	0.900	0.757(0.637‐0.878)
Random Forest	train	1.000	1.000	1.000
	test	0.562	0.933	0.765(0.645‐0.884)
XGBoost	train	1.000	1.000	1.000
	test	0.719	0.700	0.766(0.646‐0.885)

**TABLE 6:** The results of brian atrophy feature to predict the execution of 5 machine learning algorithms

**Model**	**Set**	**Sensitivity**	**Specificity**	**AUC(95%CI)**
Gradient Boosting Machine	train	0.849	0.870	0.911(0.873‐0.948)
	test	0.581	0.789	0.729(0.602‐0.857)
SVM	train	0.814	0.961	0.938(0.909‐0.966)
	test	0.791	0.579	0.710(0.572‐0.848)
Regularized Logistic Regression	train	0.797	0.714	0.817(0.763‐0.872)
	test	0.605	0.895	0.742(0.612‐0.872)
Random Forest	train	1.000	1.000	1.000
	test	0.698	0.684	0.700(0.552‐0.848)
XGBoost	train	1.000	1.000	1.000
	test	0.605	0.789	0.692(0.545‐0.838)

**TABLE 7:** The results of brian atrophy feature to predict the visuospatial of 5 machine learning algorithms

**Model**	**Set**	**Sensitivity**	**Specificity**	**AUC(95%CI)**
Gradient Boosting Machine	train	0.842	0.887	0.913(0.878‐0.949)
	test	0.681	0.733	0.691(0.540‐0.841)
SVM	train	0.911	0.935	0.959(0.928‐0.990)
	test	0.255	1.000	0.657(0.505‐0.809)
Regularized Logistic Regression	train	0.626	0.839	0.794(0.734‐0.854)
	test	0.533	0.933	0.719(0.588‐0.851)
Random Forest	train	1.000	1.000	1.000
	test	0.383	1.000	0.667(0.525‐0.810)
XGBoost	train	1.000	1.000	1.000
	test	0.319	1.000	0.627(0.475‐0.779)

## References

[brb32726-bib-0001] Alzheimer's Association Report. (2021). Alzheimer's disease facts and figures. Alzheimer's & Dementia, 17, 327–406. 10.1002/alz.12328 33756057

[brb32726-bib-0002] Aljondi, R. , Szoeke, C. , Steward, C. , Yates, P. , & Desmond, P. (2019). A decade of changes in brain volume and cognition. Brain Imaging and Behavior, 13, 554–563. 29744801 10.1007/s11682-018-9887-z

[brb32726-bib-0003] Brayne, C. , Ince, P. G. , Keage, H. A. D. , Mckeith, I. G. , Matthews, F. E. , Polvikoski, T. , & Sulkava, R. (2010). Education, the brain and dementia: Neuroprotection or compensation? Brain, 133, 2210–2216. 20826429 10.1093/brain/awq185

[brb32726-bib-0004] Cho, H. , Kwon, J.‐H. , & Seo, H.‐J. (2009). Medial temporal lobe atrophy in vascular dementia: Visual temporal lobe rating scale. Archives of Gerontology and Geriatrics, 48, 415–418. 18468705 10.1016/j.archger.2008.03.014

[brb32726-bib-0005] Chrzan, R. , Gleå, A. , Bryll, A. , & Urbanik, A. (2019). Computed tomography assessment of brain atrophy in centenarians. International Journal of Environmental Research and Public Health, 16, 3659. 10.3390/ijerph16193659 PMC6801833 31569457

[brb32726-bib-0006] Del Brutto, O. H. , Mera, R. M. , Zambrano, M. , Soriano, F. , & Lama, J. (2015). Global cortical atrophy (GCA) associates with worse performance in the Montreal Cognitive Assessment (MoCA). A population‐based study in community‐dwelling elders living in rural Ecuador. Archives of Gerontology and Geriatrics, 60, 206–209. 25306507 10.1016/j.archger.2014.09.010

[brb32726-bib-0007] Deo, R. C. (2015). Machine learning in medicine. Circulation, 132, 1920–1930. 26572668 10.1161/CIRCULATIONAHA.115.001593 PMC5831252

[brb32726-bib-0008] Emre, M. , Aarsland, D. , Brown, R. , Burn, D. J. , Duyckaerts, C. , Mizuno, Y. , Broe, G. A. , Cummings, J. , Dickson, D. W. , Gauthier, S. , Goldman, J. , Goetz, C. , Korczyn, A. , Lees, A. , Levy, R. , Litvan, I. , Mckeith, I. , Olanow, W. , Poewe, W. , … Dubois, B. (2007). Clinical diagnostic criteria for dementia associated with Parkinson's disease. Movement Disorders, 22, 1689–1707. quiz 1837. 17542011 10.1002/mds.21507

[brb32726-bib-0009] Frenzel, S. , Wittfeld, K. , Habes, M. , Klinger‐König, J. , Bülow, R. , Völzke, H. , & Grabe, H. J. (2019). A biomarker for Alzheimer's disease based on patterns of regional brain atrophy. Front Psychiatry, 10, 953. 31992998 10.3389/fpsyt.2019.00953 PMC6970941

[brb32726-bib-0010] Gorno‐Tempini, M. L. , Hillis, A. E. , Weintraub, S. , Kertesz, A. , Mendez, M. , Cappa, S. F. , Ogar, J. M. , Rohrer, J. D. , Black, S. , Boeve, B. F. , Manes, F. , Dronkers, N. F. , Vandenberghe, R. , Rascovsky, K. , Patterson, K. , Miller, B. L. , Knopman, D. S. , Hodges, J. R. , Mesulam, M. M. , & Grossman, M. (2011). Classification of primary progressive aphasia and its variants. Neurology, 76, 1006–1014. 21325651 10.1212/WNL.0b013e31821103e6 PMC3059138

[brb32726-bib-0011] Grassi, M. , Perna, G. , Caldirola, D. , Schruers, K. , Duara, R. , & Loewenstein, D. A. (2018). A clinically‐translatable machine learning algorithm for the prediction of Alzheimer's disease conversion in individuals with mild and premild cognitive impairment. Journal of Alzheimer's Disease, 61, 1555–1573. 10.3233/JAD-170547 PMC6326743 29355115

[brb32726-bib-0012] Hou, X.‐H. , Feng, L. , Zhang, C. , Cao, X.‐P. , Tan, L. , & Yu, J.‐. T. (2019). Models for predicting risk of dementia: A systematic review. Journal of Neurology, Neurosurgery, and Psychiatry, 90, 373–379. 29954871 10.1136/jnnp-2018-318212

[brb32726-bib-0013] Jokinen, H. , Koikkalainen, J. , Laakso, H. M. , Melkas, S. , Nieminen, T. , Brander, A. , Korvenoja, A. , Rueckert, D. , Barkhof, F. , Scheltens, P. , Schmidt, R. , Fazekas, F. , Madureira, S. , Verdelho, A. , Wallin, A. , Wahlund, L.‐. O. , Waldemar, G. , Chabriat, H. , Hennerici, M. , … Erkinjuntti, T. (2020). Global burden of small vessel disease related brain changes on MRI predicts cognitive and functional decline. Stroke; A Journal of Cerebral Circulation, 51, 170–178. 10.1161/STROKEAHA.119.026170 PMC6924941 31699021

[brb32726-bib-0014] Jokinen, H. , Lipsanen, J. , Schmidt, R. , Fazekas, F. , Gouw, A. A. , Van Der Flier, W. M. , Barkhof, F. , Madureira, S. , Verdelho, A. , Ferro, J. M. , Wallin, A. , Pantoni, L. , Inzitari, D. , & Erkinjuntti, T. (2012). Brain atrophy accelerates cognitive decline in cerebral small vessel disease: The LADIS study. Neurology, 78, 1785–1792. 22592361 10.1212/WNL.0b013e3182583070

[brb32726-bib-0015] Kivipelto, M. , Ngandu, T. , Laatikainen, T. , Winblad, B. , Soininen, H. , & Tuomilehto, J. (2006). Risk score for the prediction of dementia risk in 20 years among middle aged people: A longitudinal, population‐based study. Lancet Neurology, 5, 735–741. 16914401 10.1016/S1474-4422(06)70537-3

[brb32726-bib-0016] Koedam, E. L. G. E. , Lehmann, M. , Van Der Flier, W. M. , Scheltens, P. , Pijnenburg, Y. A. L. , Fox, N. , Barkhof, F. , & Wattjes, M. P. (2011). Visual assessment of posterior atrophy development of a MRI rating scale. European Radiology, 21, 2618–2625. 21805370 10.1007/s00330-011-2205-4 PMC3217148

[brb32726-bib-0017] Lee, J. S. , Kim, C. , Shin, J.‐. H. , Cho, H. , Shin, D.‐. S. , Kim, N. , Kim, H. J. , Kim, Y. , Lockhart, S. N. , Na, D. L. , Seo, S. W. , & Seong, J.‐. K. (2018). Machine learning‐based individual assessment of cortical atrophy pattern in Alzheimer's disease spectrum: Development of the classifier and longitudinal evaluation. Science Reports, 8, 4161. 10.1038/s41598-018-22277-x PMC5841386 29515131

[brb32726-bib-0018] Li, C. H. , Fan, S. P. , Chen, T. F. , Chiu, M. J. , Yen, R. F. , & Lin, C. H. (2020). Frontal variant of Alzheimer's disease with asymmetric presentation mimicking frontotemporal dementia: Case report and literature review. Brain and Behavior, 10, e01548. 31989779 10.1002/brb3.1548 PMC7066333

[brb32726-bib-0019] Li, H. , Jia, J. , & Yang, Z. (2016). Mini‐mental state examination in elderly Chinese: A population‐based normative study. Journal of Alzheimer's Disease, 53, 487–496. 10.3233/JAD-160119 27163822

[brb32726-bib-0020] Luo, W. , Phung, D. , Tran, T. , Gupta, S. , Rana, S. , Karmakar, C. , Shilton, A. , Yearwood, J. , Dimitrova, N. , Ho, T. B. , Venkatesh, S. , & Berk, M. (2016). Guidelines for developing and reporting machine learning predictive models in biomedical research: A multidisciplinary view. Journal of Medical Internet Research [Electronic Resource], 18, e323. 10.2196/jmir.5870 PMC5238707 27986644

[brb32726-bib-0021] McInnes, K. , Friesen, C. L. , Mackenzie, D. E. , Westwood, D. A. , & Boe, S. G. (2017). Mild traumatic brain injury (mTBI) and chronic cognitive impairment: A scoping review. Plos One, 12, e0174847. 28399158 10.1371/journal.pone.0174847 PMC5388340

[brb32726-bib-0022] McKhann, G. M. , Knopman, D. S. , Chertkow, H. , Hyman, B. T. , Jack, C. R. , Kawas, C. H. , Klunk, W. E. , Koroshetz, W. J. , Manly, J. J. , Mayeux, R. , Mohs, R. C. , Morris, J. C. , Rossor, M. N. , Scheltens, P. , Carrillo, M. C. , Thies, B. , Weintraub, S. , & Phelps, C. H. (2011). The diagnosis of dementia due to Alzheimer's disease: Recommendations from the National Institute on Aging–Alzheimer's Association workgroups on diagnostic guidelines for Alzheimer's disease. Alzheimer's & Dementia, 7, 263–269. 10.1016/j.jalz.2011.03.005 PMC3312024 21514250

[brb32726-bib-0023] Meguro, K. , Constans, J.‐. M. , Shimada, M. , Yamaguchi, S. , Ishizaki, J. , Ishii, H. , Yamadori, A. , & Sekita, Y. (2003). Corpus callosum atrophy, white matter lesions, and frontal executive dysfunction in normal aging and Alzheimer's Disease. A community‐based study: The Tajiri project. International Psychogeriatrics, 15, 9–25. 12834197 10.1017/s104161020300872x

[brb32726-bib-0024] Oosterman, J. M. , Oosterveld, S. , Rikkert, M. G. O. , Claassen, J. A. , & Kessels, R. P. C. (2012). Medial temporal lobe atrophy relates to executive dysfunction in Alzheimer's disease. International Psychogeriatrics, 24, 1474–1482. 22717328 10.1017/S1041610212000506

[brb32726-bib-0025] Pleizier, C. M. , Van Der Vlies, A. E. , Koedam, E. , Koene, T. , Barkhof, F. , Van Der Flier, W. M. , Scheltens, P. , & Pijnenburg, Y. (2012). Episodic memory and the medial temporal lobe: Not all it seems. Evidence from the temporal variants of frontotemporal dementia. Journal of Neurology, Neurosurgery, and Psychiatry, 83, 1145–1148. 22933816 10.1136/jnnp-2012-302437

[brb32726-bib-0026] Poulakis, K. , Pereira, J. B. , Mecocci, P. , Vellas, B. , Tsolaki, M. , KÅ‚Oszewska, I. , Soininen, H. , Lovestone, S. , Simmons, A. , Wahlund, L.‐O. , & Westman, E. (2018). Heterogeneous patterns of brain atrophy in Alzheimer's disease. Neurobiology of Aging, 65, 98–108. 29455029 10.1016/j.neurobiolaging.2018.01.009

[brb32726-bib-0027] Reith, W. , & Mühl‐Benninghaus, R. (2015). Differential diagnostics of dementia type diseases. Radiology, 55, 378–385. 10.1007/s00117-014-2799-z 25944275

[brb32726-bib-0028] Sachdev, P. , Kalaria, R. , Oâ™Brien, J. , Skoog, I. , Alladi, S. , Black, S. E. , Blacker, D. , Blazer, D. G. , Chen, C. , Chui, H. , Ganguli, M. , Jellinger, K. , Jeste, D. V. , Pasquier, F. , Paulsen, J. , Prins, N. , Rockwood, K. , Roman, G. , & Scheltens, P. (2014). Diagnostic criteria for vascular cognitive disorders. Alzheimer Disease and Associated Disorders, 28, 206–218. 24632990 10.1097/WAD.0000000000000034 PMC4139434

[brb32726-bib-0029] Sacuiu, S. , Eckerström, M. , Johansson, L. , Kern, S. , Sigström, R. , Xinxin, G. , Östling, S. , & Skoog, I. (2018). Increased risk of dementia in subjective cognitive decline if CT brain changes are present. Journal of Alzheimer's Disease, 66, 483–495. 10.3233/JAD-180073 PMC6218129 30320572

[brb32726-bib-0030] Savarraj, J. P. J. , Kitagawa, R. , Kim, D. H. , & Choi, H. A. (2021). White matter connectivity for early prediction of Alzheimer's disease. Technology and Health Care, 30(1), 17–28. 10.3233/THC-192012 33998562

[brb32726-bib-0031] Shigemizu, D. , Akiyama, S. , Asanomi, Y. , Boroevich, K. A. , Sharma, A. , Tsunoda, T. , Matsukuma, K. , Ichikawa, M. , Sudo, H. , Takizawa, S. , Sakurai, T. , Ozaki, K. , Ochiya, T. , & Niida, S. (2019). Risk prediction models for dementia constructed by supervised principal component analysis using miRNA expression data. Communications Biology, 2, 77. 30820472 10.1038/s42003-019-0324-7 PMC6389908

[brb32726-bib-0032] Shimoda, A. , Li, Y. , Hayashi, H. , & Kondo, N. (2021). Dementia risks identified by vocal features via telephone conversations: A novel machine learning prediction model. Plos One, 16, e0253988. 34260593 10.1371/journal.pone.0253988 PMC8279312

[brb32726-bib-0033] Skrobot, O. A. , O'Brien, J. , Black, S. , Chen, C. , Decarli, C. , Erkinjuntti, T. , Ford, G. A. , Kalaria, R. N. , Pantoni, L. , Pasquier, F. , Roman, G. C. , Wallin, A. , Sachdev, P. , Skoog, I. , Taragano, F. E. , Kril, J. , Cavalieri, M. , Jellinger, K. A. , Kovacs, G. G. , … Kehoe, P. G. (2017). The vascular impairment of cognition classification consensus study. Alzheimer's & Dementia, 13, 624–633. 10.1016/j.jalz.2016.10.007 27960092

[brb32726-bib-0034] Stern, Y. (2006). Cognitive reserve and Alzheimer disease. Alzheimer Disease and Associated Disorders, 20, S69–S74. 16917199 10.1097/00002093-200607001-00010

[brb32726-bib-0035] Stoub, T. R. , Rogalski, E. J. , Leurgans, S. , Bennett, D. A. , & Detoledo‐Morrell, L. (2010). Rate of entorhinal and hippocampal atrophy in incipient and mild AD: Relation to memory function. Neurobiology of Aging, 31, 1089–1098. 18809228 10.1016/j.neurobiolaging.2008.08.003 PMC2873053

[brb32726-bib-0036] Soderlund, H. , Nyberg, L. , & Nilsson, L.‐G. (2004). Cerebral atrophy as predictor of cognitive function in old, community‐dwelling individuals. Acta Neurologica Scandinavica, 109, 398–406. 15147463 10.1111/j.1600-0404.2004.00239.x

[brb32726-bib-0037] Tam, A. , Dansereau, C. , Iturria‐Medina, Y. , Urchs, S. , Orban, P. , Sharmarke, H. , Breitner, J. , & Bellec, P. (2019). A highly predictive signature of cognition and brain atrophy for progression to Alzheimer's dementia. Gigascience, 8, giz055. 31077314 10.1093/gigascience/giz055 PMC6511068

[brb32726-bib-0038] Tondelli, M. , Wilcock, G. K. , Nichelli, P. , De Jager, C. A. , Jenkinson, M. , & Zamboni, G. (2012). Structural MRI changes detectable up to ten years before clinical Alzheimer's disease. Neurobiology of Aging, 33, 825.e25–S36. 10.1016/j.neurobiolaging.2011.05.018 21782287

[brb32726-bib-0039] Uribe‐San‐Martín, R. , Ciampi, E. , Di Giacomo, R. , VÃ¡Squez, M. , CÃ¡Rcamo, C. , Godoy, J. , Lo Russo, G. , & Tassi, L. (2018). Corpus callosum atrophy and post‐surgical seizures in temporal lobe epilepsy associated with hippocampal sclerosis. Epilepsy Research, 142, 29–35. 29549794 10.1016/j.eplepsyres.2018.03.001

[brb32726-bib-0040] Wang, X. , Zhu, T. , Xia, M. , Liu, Y. u. , Wang, Y. , Wang, X. , Zhuang, L. , Zhong, D. , Zhu, J. , He, H. , Weng, S. , Zhu, J. , & Lai, D. (2022). Predicting the prognosis of patients in the coronary care unit: A novel multi‐category machine learning model using XGBoost. Frontiers in Cardiovascular Medicine, 9, 764629. 35647052 10.3389/fcvm.2022.764629 PMC9133425

[brb32726-bib-0041] Wilson, R. S. , Yu, L. , Lamar, M. , Schneider, J. A. , Boyle, P. A. , & Bennett, D. A. (2019). Education and cognitive reserve in old age. Neurology, 92, e1041–e1050. 30728309 10.1212/WNL.0000000000007036 PMC6442015

[brb32726-bib-0042] Zhang, B. , Lin, L. , & Wu, S. (2021). A review of brain atrophy subtypes definition and analysis for Alzheimer's disease heterogeneity studies. Journal of Alzheimer's Disease, 80, 1339–1352. 10.3233/JAD-201274 33682711

[brb32726-bib-0043] Zhang, L. , Sun, W.‐. H. , Xing, M. , Wang, Y. , Zhang, Y. , Sun, Q. , Cheng, Y. , Shi, C. , & Zhang, N. (2019). Medial temporal lobe atrophy is related to learning strategy changes in amnestic mild cognitive impairment. Journal of the International Neuropsychological Society, 25, 706–717. 31023395 10.1017/S1355617719000353

[brb32726-bib-0044] Zhang, Y. , Londos, E. , Minthon, L. , Wattmo, C. , Liu, H. , Aspelin, P. , & Wahlund, L.‐O. (2008). Usefulness of computed tomography linear measurements in diagnosing Alzheimer's disease. Acta Radiologica, 49, 91–97. 18210318 10.1080/02841850701753706

